# Raddeanin A (RA) reduced acute inflammatory injury in mouse experimental cerebral hemorrhage by suppression of TLR4

**DOI:** 10.7150/ijms.73007

**Published:** 2022-07-11

**Authors:** Bo Hei, Jia Ouyang, Jingru Zhou, Dongliang Wang, Zeyu Miao, Ru-en Liu

**Affiliations:** Department of neurosurgery, Peking University People's Hospital, Peking University, Beijing, China

**Keywords:** Intracerebral hemorrhage, Raddeanin A, inflammation, microglia, TLR4

## Abstract

Spontaneous intracerebral hemorrhage (ICH) is associated with high mortality and disability rates. The microglia-induced inflammatory response is a critical factor determining brain tissue damage after ICH. Raddeanin A (RA) is a natural triterpenoid compound with anti-inflammatory effects, although its effects on ICH and the underlying molecular mechanism have not been elucidated. In this study, we found that RA reduced the volume of cerebral hematoma and cerebral edema, attenuated neuronal apoptosis and improved the behavioral indices in a murine model of acute cerebral hemorrhage. Mechanistically, RA downregulated the TLR4-mediated pro-inflammatory effectors, reduced infiltration of microglia in peri-intracerebral hemorrhage and inhibited apoptosis of neurons co-cultured with activated microglia. In conclusion, RA can alleviate ICH-related tissue damage and promote the recovery of neuronal function by suppressing microglia-induced inflammation and apoptosis.

## Introduction

Intracerebral hemorrhage (ICH) is the result of ruptured vessels due to trauma or chronic hypertension. It is associated with high rates of morbidity and mortality, and the incidence rate is 12-15/100,000 person-years. Cerebral hemorrhage accounts for about 15% of all strokes and 10-30% of strokes in hospitalized patients in Western countries, and 18.8% to 47.6% of strokes in China. The mortality rate within 30 days of onset is as high as 35% to 52%, and only 20% of the patients recover their ability to perform daily activities after 6 months, resulting in considerable social and economic burden 1, 2. The existing neuroprotective drugs cannot effectively improve patient survival or quality of life after ICH. Therefore, it is crucial to explore the pathological mechanisms of ICH in order to develop novel safe and effective treatment options.

The presence of extravasated blood due to vessel rupture triggers microglial activation and inflammation. Multiple studies have confirmed that microglia-mediated inflammatory respond plays a crucial role in brain damage following ICH. Activated microglia infiltrate into the peri-hemorrhagic regions and secrete high levels of inflammatory cytokines such as tumor necrosis factor-α (TNF-α) and interleukin-1β (IL-1β), along with free radicals and chemokines, which result in neuronal apoptosis and exacerbate cerebral edema 3. Thus, attenuating microglial activation can be an effective therapeutic strategy against ICH 4, 5. Microglia activation is initiated by the toll-like receptor 4 (TLR4), which activates downstream inflammatory pathways and promotes secretion of cytokines and other effectors. Inhibition of TLR4-mediated inflammation is therefore a promising treatment strategy for ICH.

Raddeanin A(RA), an oleanane type triterpenoid, is a major bioactive compound isolated from *Anemone raddeana* Regel^6^. It exhibits anti-tumor effects against non-small cell lung cancer, glioblastoma, cholangiocarcinoma and so on 7-10. In addition, RA also exerts antioxidant effects and mitigates inflammation via the NF-κB signaling pathway 11, 12. The aim of this study was to analyze the effects of RA on acute ICH and explore the underlying mechanisms. We found that RA can alleviate the symptoms of ICH by mitigating the TLR4-mediated inflammatory response.

## Methods and Materials

### Chemicals, reagents and antibodies

Puruifa Biotechnology Co. Ltd (Chengdu, China) provided RA, which was dissolved in cell culture grade dimethyl sulfoxide (DMSO) and stored at 4°C. Dulbecco's modified Eagle medium (DMEM) and fetal bovine serum (FBS) were from Gibco (Grand Island, USA). Antibodies against TLR4, TNF, IL-1, iNOS and GAPDH were bought from Cell Signaling Technology (Beverly, MA).

### Establishment of ICH model

A total of 180 eight to ten weeks old male C57BL/6 mice weighing 25-35 g (8-10 weeks old) were used in the experiments and the mice were provided by the Animal Center at Huafukang (Beijing, China). All animal procedures were conducted in compliance with the guidelines of the Army General Hospital's Committee on the Ethics of Animal Experiments, and reported using the ARRIVE criteria. Throughout the study, the mice had unrestricted access to food and water. ICH was induced with collagenase injection as reported previously. Briefly, the mice were anesthetized with isoflurane (3% for induction, 1% for maintenance) and ventilated with oxygen-enriched air (20%:80%) via a nasal cone. To induce bleeding, bacterial collagenase VII (0.1 U in 0.4μl; Sigma) was injected into the right caudate at the following stereotactic coordinates: 0.5 mm anterior and 2.0 mm lateral of the bregma, 2.8 mm in depth. Collagenase was administered over a 5-minute period, and the needle was kept in place for another 15 minutes to prevent reflux. The mice/10g were intraperitoneally injected with 100μL RA or an equal amount of vehicle (DMSO concentration <1%) 15 minutes after ICH.

### Neurological Scoring

Two trained investigators who were blinded to the experimental groups assessed the neurological deficit in the mice 24, 48 and 72h post-ICH using the Modified Neurological Severity Score (mNSS) (each group n = 180). The severity of neurological impairment was graded on a scale of 0 to 18, with 0 indicating normal function and 18 indicating impaired function.

### Assessment of lesion volume

Three days following ICH, the mouse brains were sliced coronally at the needle entry site to acquire 1 mm-thick serial slices (1 mm thick) anterior and posterior to the needle entrance plane (each group n = 5). Picture-Pro Plus software (Media Cybernetics, California, USA) was used to image the tissue slices, and the lesion volume was estimated by adding the lesioned areas of all slices.

### Assessment of brain edema

The brain water content (each group n = 5) was measured 72 hours after ICH using the wet-dry method described previously with the following formula: water content = (wet weight - dried weight)/wet weight.

### Reverse transcription-quantitative PCR (RT-qPCR)

Cells and tissues (the used brain tissue was from the 1mm around the brain hemorrhage, each group n = 5) RNA was isolated using TRIzol reagent (Invitrogen; Thermo Fisher Scientific, Inc.), and reverse transcribed to cDNA using high-capacity RNA-to-cDNA kit (Applied Biosystems). RT-PCR was performed using the 2x Fast SYBR Green master mix on the ABI 7000 Sequence Detection System (Applied Biosystems).

### Western blotting

Cells and tissues (The used brain tissue was from the 1mm around the brain hemorrhage) were lysed in RIPA buffer (CWBio, Beijing, China), and 30g lysate per sample was separated on 10% SDS-PAGE gels and transferred onto a polyvinylidene difluoride (PVDF) membrane (Hyclone Laboratories, Logan, USA) (each group n = 5). After blocking for 1 hour (5% non-fat milk dissolved in TBS-Tween-20), the membranes were incubated overnight with primary antibodies and then with HRP-conjugated secondary antibodies at 4°C. ECL luminescence reagents were used to detect the positive bands.

### Immunofluorescence

Mice brain tissue (each group n = 5) sections (15μm thick) and cultured cells were blocked with goat serum, incubated overnight with primary antibodies at 4°C, washed, and exposed to secondary antibodies for 1 hour at 25°C. After counterstaining with diamidino-2-phenylindole (DAPI;0.5 lg/mL; Roche, Indianapolis, IN), the stained samples were observed under a fluorescence microscope.

### TUNEL staining

TUNEL staining (each group n = 5) was performed using the In Situ Cell Death Detection Kit of Roche Molecular Biochemicals (Mannheim, Germany) according to the manufacturer's instructions. The stained sections were observed under a fluorescence microscope (Leica, Hamburg, Germany), and the positive cells in the cortex around the hematoma were counted by three researchers blinded to the grouping.

### Primary neuron culture

Pregnant mice were humanely euthanized on post-coital days 16-18, and the embryonic cortical neurons were dissociated by incubating with accutase containing 100U/ml DNAse I for 20 minutes at 37°C. The enzyme solution was discarded and the tissues were rinsed in warmed DMEM containing 20% FBS. The digested mass was passed through a mesh to obtain single cells, which were then plated on poly-D-lysine-coated 24-well plates at the density of 20000 cells/well. The culture media was replaced with NeuroBasal medium (Invitrogen, MA, USA) with 2% B27 (Invitrogen) and 1% GlutaMAX-I after overnight incubation (Invitrogen). The neurons were cultivated for three days at 37°C with 5% CO_2_, and then Ara-C was added to prevent non-neuronal cells from proliferating. The cells were harvested after 14 days of culture.

### Transwell co-culture

The primary neurons and microglial cells were co-cultured using Transwell inserts. Briefly, BV2 cells were seeded in the upper chamber at the density of 2 × 10^4^ cells/well, and the primary neurons were seeded in the lower chamber at the density of 1 × 10^4^ cells/well in neurobasal medium supplemented with 2% B27 and 1% glutamine. The cells were cultured at 37°C under 5% CO_2_.

### Statistical analyses

The results were expressed as the mean ± SD of at least three independent experiments.

Statistical analysis was performed with SPSS 20.0. One-Way ANOVA was used for the multiple group comparison followed by LSD or Dunnett's post hoc test and p < 0.05 was considered statistically significant.

## Results

### RA alleviated the neurological and pathological damage associated with ICH

**Figure 1:**
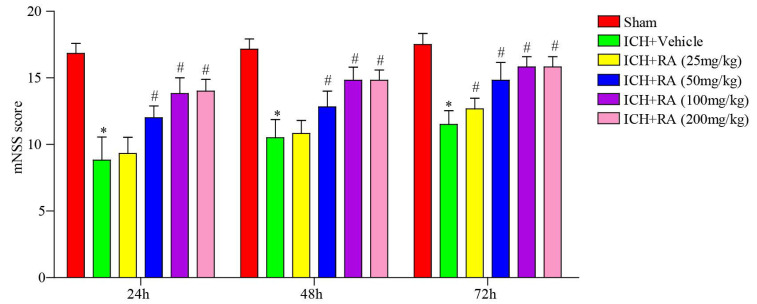
** RA reduced the neurological deficits induced by ICH.** mNSS scores of different groups at the indicated time point after ICH induction or sham-operation. *p<0.05, **p<0.01, compared to sham group; # p<0.05, compared to ICH + vehicle group. Each group n=5.

The therapeutic effect of different doses of RA on the mouse model of ICH was evaluated in terms of neurobehavioral function. As shown in Figure 1, RA treatment significantly improved the mNSS in the ICH mice in a time- and dose-dependent manner. RA was ineffective at doses less than 25 mg/kg, whereas doses higher than 100 mg/kg did not confer any additional benefit. The optimum dosage of RA was 100 mg/kg, and therefore 50 mg/kg and 100 mg/kg RA were used in subsequent experiments.

**Figure 2:**
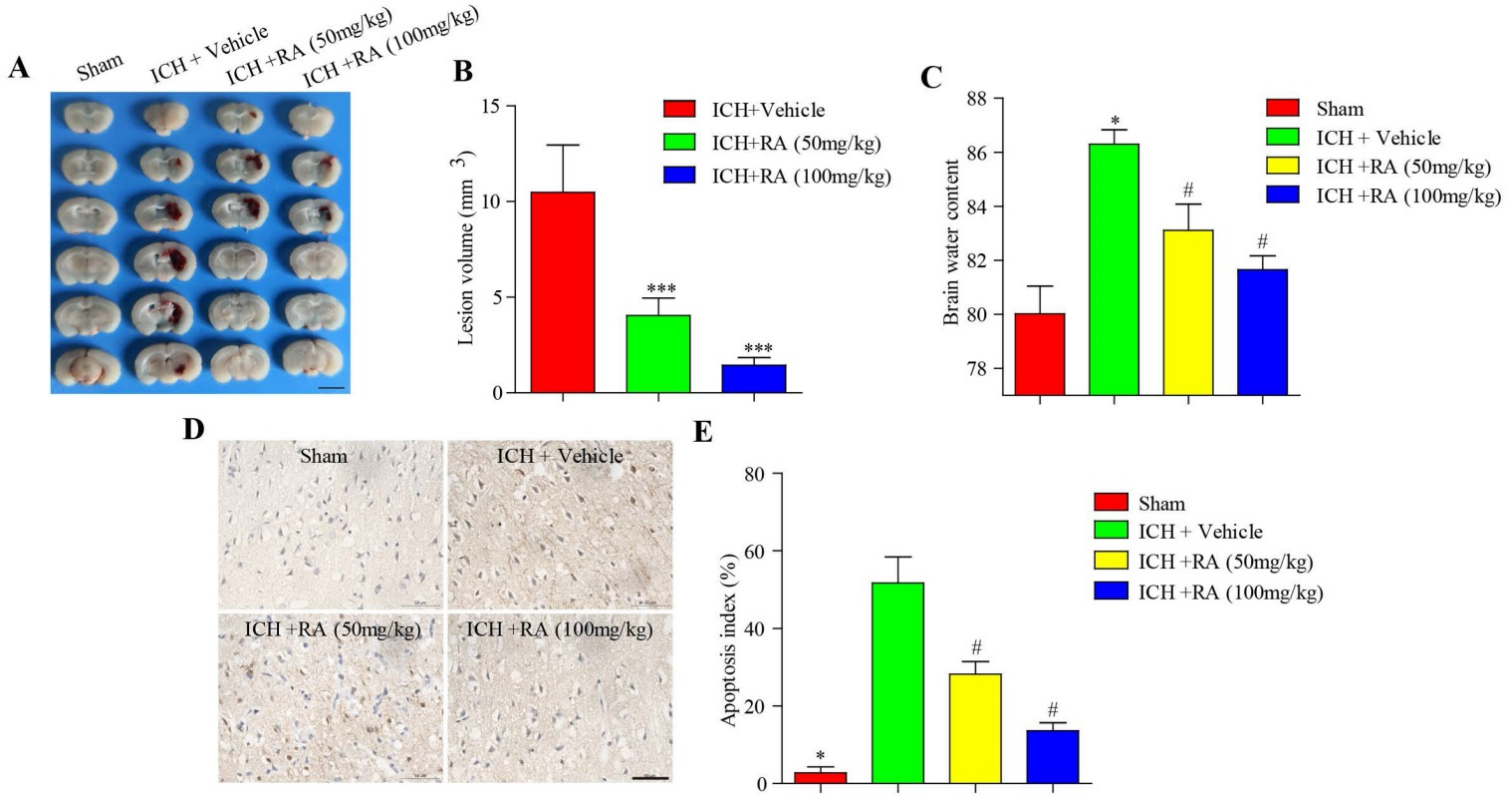
** RA alleviated the pathological deficits of ICH. (A)** Representative images of brain sections showing the lesion areas (Bar = 5mm). **(B)** Quantification of the lesion volume 72h post-ICH. **(C)** Brain water content of the ipsilateral hemorrhagic hemispheres in the different groups. *p<0.05, **p<0.01, compared to sham group; # p<0.05, compared to ICH + vehicle group. **(D)** Percentage of apoptotic cells after 72h (Bar = 50μm).

Consistent with the changes in neurological scores, RA dramatically reduced the volume of intracerebral lesions post-ICH in a dose-dependent manner (Figure 2A-B). The brain water level increased considerably after ICH compared to that in the sham-operated mice, and was reduced in a dose-dependent manner by RA (Figure 2C). Finally, RA also reduced the degree of apoptosis in and around the hemorrhagic lesions (Figure 2D-E). Taken together, RA protected mice from ICH-induced neuropathological deterioration.

### RA attenuated microglial infiltration and TLR4-mediated inflammation in the peri-hemorrhagic areas

**Figure 3:**
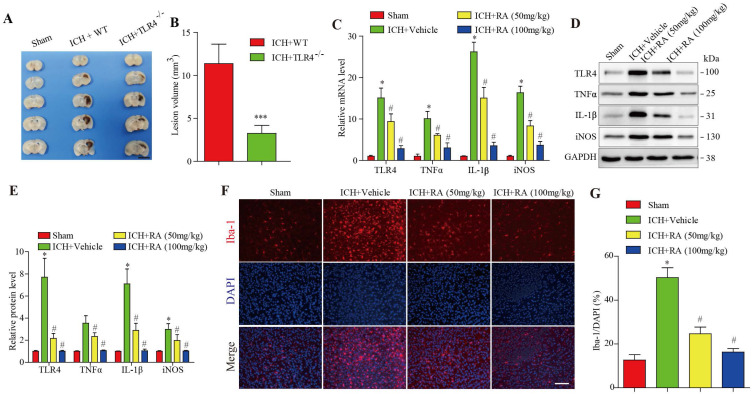
**RA attenuated TLR4 signaling and microglial activation in peri-hemorrhagic areas. (A-B)** TLR4-deficient reduced hemorrhagic lesions in mice ICH model (A) Representative images of brain sections showing the lesion areas (Bar = 5mm) (B) Quantification of the lesion volume 72h post-ICH. ***p<0.01, compared to ICH +WT group. Each group n=5. **(C)** RT-PCR results and **(D-E)** immunoblots showing the mRNA and protein levels of TLR4 and its downstream effectors 72h after ICH induction. **(F-G)** Representative images showing iba-1+ active microglial cells in the lesions and its quantification after 72h (scale bar = 50μm). *p<0.05, compared to sham group; # p<0.05, compared to ICH + vehicle group.

Compared with the wild type mice, after 72 hours of collagenase-induced ICH, the hemorrhagic lesions in TLR4^-/-^ mice brain were less (Figure 2A-B). Given the pathological role of the TLR4 signaling pathway in ICH, we analyzed the levels of TLR4 and its downstream effectors in the hemorrhagic lesions. After 72 hours of collagenase-induced ICH, the brain tissues showed a marked increase in TLR4 mRNA and protein levels, as well as pro-inflammatory factors such as TNF-α, IL-1β, and iNOS. RA treatment significantly downregulated TLR4 and its downstream effectors in a dose-dependent manner (Figure 3C-E). Furthermore, RA also reduced the infiltration of microglial cells in the ICH lesions in a dose-dependent manner (Figure 3F-G). These data suggested that RA mitigated ICH-induced inflammation by targeting the TLR4 signaling pathway.

### RA protected neurons against apoptosis and inflammatory damage induced by RBC lysis and microglial activation

**Figure 4:**
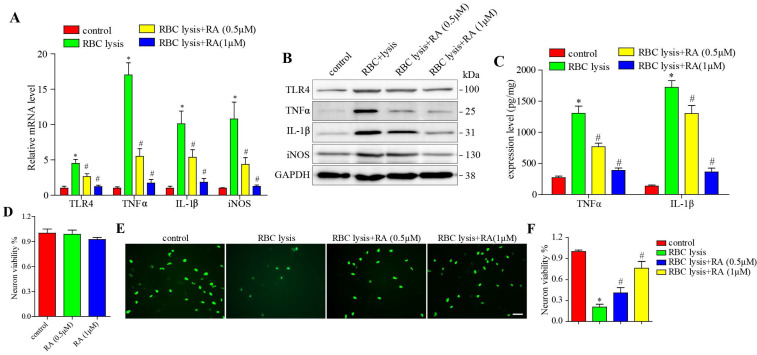
**RA reduced microglial activation and neuronal damage induced by RBC lysis *in vitro*. (A)** RT-PCR results and **(B)** immunoblots showing the mRNA and protein levels of TLR4 and its downstream effectors as well as **(C)** ELISA showing the expression level of TNFα and IL-1β in microglial cells co-cultured with primary neurons in the presence of RBC lysates and treated with RA. **(D)** The effects of RA alone on microglia and primary neurons co-cultured system. CCK8 assay was used to detect the neuronal viability. **(E-F)** Representative images of Neun+ (green) primary neurons co-cultured and treated as described (scale bar = 50μm) and the percentage of viable cells. *p<0.05, compared to sham group; # p<0.05, compared to ICH + vehicle group.

To validate the above hypothesis, we stimulated microglial cells with murine RBC lysates with and without RA treatment, and found that RBC lysis elevated TLR4, TNF-α, IL-1β, and iNOS levels in a dose-dependent manner, which was blocked by RA (Figure 4A-B). Next, we detected the released level of TNF-α and IL-1β, consisting with the above results, RBC lysis increased TNF-α and IL-1β release levels in a dose-dependent manner, which was reduced by RA (Figure 4C). We then established a co-culture of microglia and neurons, and found that RA alone has no effects on neuronal viability (Figure 4D) and RBC lysis reduced the number of neurons, which was restored by RA in a dose-dependent manner (Figure 4E-F). Furthermore, RA inhibited the TLR4 pathway in the microglia and protected the neurons from inflammatory damage.

## Discussion

ICH refers to non-traumatic hemorrhage caused by rupture of blood vessels in the cerebral parenchyma, and is associated with high mortality and disability rates due to lack of effective treatment options. The secondary injuries resulting from cerebral hemorrhage, including cerebral hematoma and edema, further exacerbate neuronal apoptosis^13^, ^14^. RA is an active triterpenoid saponin extracted from *A. raddeana* Regel, and has potent anti-tumor effects^6^. In this study, RA significantly improved neurological scores and decreased ICH-induced cerebral hematoma and edema in a mouse model. Mechanistically, RA reduced microglial infiltration and the ensuing inflammatory response and neuronal apoptosis.

Inflammation is the key pathological basis of ICH, which leads to mitochondrial damage and excessive cytokine production, eventually progressing to neuronal apoptosis and aberrant sensorimotor function 15. Microglia are the major inflammatory cells in neurological tissues, and inhibiting microglial infiltration and the ensuing inflammatory response is an effective therapeutic strategy against ICH 16, 17. Several natural compounds are known to alleviate the symptoms of ICH by suppressing inflammation 17-19. In addition, we previously found that RA inhibited human colorectal cancer and osteosarcoma cell growth by downregulating the pro-inflammatory NF-κB signaling pathway. This further underscores the mechanistic role of the anti-inflammatory effect of RA in improving the neurological defects and neuronal apoptosis associated with ICH.

TLR4 activation has been linked to excessive cytokine production and microglial activation, which contribute to ICH-related inflammatory damage. TLR4 mRNA and protein expression levels were significantly elevated in the murine ICH model, which corresponded to increased levels of the pro-inflammatory cytokines TNF-α and IL-1β 20, 21. However, RA treatment markedly reduced the inflammatory effectors. In addition, RA also downregulated TLR4 expression and blocked pro-inflammatory cytokine release induced by RBC lysates *in vitro* and *in vivo*. These findings suggested that the anti-inflammatory effects of RA in the ICH model are mediated via the TLR4 signaling pathway.

In conclusion, RA treatment may have therapeutic potential against brain tissue damage from secondary ICH injury. Furthermore, the anti-inflammatory and anti-microglial actions of RA were due in part to the inhibition of TLR4-dependent pathways. The clinical efficacy of RA should be investigated in ICH patients with secondary brain tissue damage.
